# DOA Estimation for Coprime Linear Array Based on MI-ESPRIT and Lookup Table

**DOI:** 10.3390/s18093043

**Published:** 2018-09-12

**Authors:** Weike Zhang, Xi Chen, Kaibo Cui, Tao Xie, Naichang Yuan

**Affiliations:** State Key Laboratory of Complex Electromagnetic Environment Effects on Electronics and Information System, National University of Defense Technology, Changsha 410073, China; xdwdz2010@163.com (W.Z.); sky_leader@126.com (K.C.); xietao09@nudt.edu.cn (T.X.); yuannaichang@hotmail.com (N.Y.)

**Keywords:** coprime linear array, DOA estimation, solving ambiguity, MI-ESPRIT, lookup table

## Abstract

In order to improve the angle measurement performance of a coprime linear array, this paper proposes a novel direction-of-arrival (DOA) estimation algorithm for a coprime linear array based on the multiple invariance estimation of signal parameters via rotational invariance techniques (MI-ESPRIT) and a lookup table method. The proposed algorithm does not require a spatial spectrum search and uses a lookup table to solve ambiguity, which reduces the computational complexity. To fully use the subarray elements, the DOA estimation precision is higher compared with existing algorithms. Moreover, the algorithm avoids the matching error when multiple signals exist by using the relationship between the signal subspace of two subarrays. Simulation results verify the effectiveness of the proposed algorithm.

## 1. Introduction

DOA estimation is an important problem in array signal processing, and is widely used in radar, communication, sonar, and other detection equipment [1,2,3,4,5,6]. Traditional subspace-based methods, which include the multiple signal classification (MUSIC) algorithm [7] and the estimation of signal parameters via rotational invariance techniques (ESPRIT) [8,9], have been verified as efficient estimation techniques by using the eigenvalue decomposition of the received covariance matrix. Previous studies focused on the uniform array, such as the uniform linear array, uniform circular array, etc. Many DOA estimation algorithms have been proposed [10,11,12,13]. However, as the number of array elements and array aperture are restricted in practice, the uniform array is not the optimal array structure. The sparse array has attracted considerable attention because it obtains a larger array aperture without increasing the number of array sensors, thus producing better DOA estimation performance. The earliest sparse array is the minimum redundancy array (MRA). However, MRA cannot obtain the closed form expression, which makes it impossible to apply in practice. Subsequently, Vaidyanathan proposed the nested array and coprime array [14,15]. The application of the nested array is restricted by the mutual coupling of array elements. The coprime array is a non-uniform array system whose inter-element spacing is larger than half-wavelength. The coprime array processes spatial signals in a sparser array structure, which results in good angle measurement performance. Compared with the uniform array, it has a larger array aperture when the number of array elements is the same, and fewer array sensors are required when the array aperture is the same. Additionally, the mutual coupling of array elements is reduced, which weakens the influence on the DOA estimation performance.

For the coprime linear array (CLA), two mainstream DOA estimation methods exist: the virtualization array sensor method [16,17,18,19,20] and the solving-ambiguity-based method [21,22,23,24,25,26]. In the solving-ambiguity-based method, CLA can be decomposed into two uniform linear subarrays, and then DOA can be achieved according to conventional DOA estimation algorithms [7,8,9]. This method sacrifices some degrees of freedom (DOF), that is to say, it reduces the number of signals that the CLA can detect. By adding a number of array sensors, we can improve the DOF. In addition, the virtualization array sensor method can add DOF by extending the virtual array aperture. However, this method is highly computationally complex and has demanding requirements for snapshots of the received signals, which is difficult in practical engineering applications. In order to achieve a better trade-off between the DOF and practical applications, we mainly studied the solving-ambiguity-based method in this paper.

In the solving-ambiguity-based method, the CLA is first decomposed into two uniform linear subarrays. Since inter-element spacing is larger than half-wavelength, the DOA estimation results are ambiguous. In order to obtain the true DOA, solving ambiguity must be performed. Zhou et al. [23] proposed a DOA estimation algorithm for CLA by combining the MUSIC of two subarrays for the corresponding coprime array signal processing. They obtained the DOA by searching the closest spectral peaks of two subarrays, but this method has higher computational complexity. In order to reduce the computational complexity, the reference [24] proposed a partial spectral search DOA estimation method. By using the Root-MUSIC algorithm for CLA, the reference [25] avoided spectral peak search. In the reference [26], a fast DOA estimation algorithm was proposed based on the unitary transformation technique, and the complexity was further reduced. The reference [27] proposed a fast search-free DOA estimation for CLA by using projection processing in the search for optimal DOAs. However, solving modular equations in the reference [27] required an iterative process, which still has a large computational burden.

In order to ease the limitations of the existing algorithms, this paper proposes a novel DOA estimation method for CLA based on the MI-ESPRIT and lookup table (LUT). MI-ESPRIT fully uses elements of every subarray, so the angle measurement precision is improved compared with the algorithm proposed by the reference [28], which was based on the ESPRIT algorithm. In addition, the computational complexity is reduced because the proposed method avoids spectral peak search. After obtaining the DOA estimation results of two subarrays, solving ambiguity faces the problem of traversal searching, and the computational complexity increases in the presence of multiple signals. In engineering applications, the common method of reducing computational complexity is to use the LUT. So, we use the LUT method to solve ambiguity in the CLA. Finally, by using the transformation relationship between the signal subspace of the two subarrays, the matching error among multiple signals is avoided. The proposed method is ultra-high speed, has higher DOA estimation precision, no matching error, and low memory usage, rendering it suitable for engineering applications.

The remainder of this paper is organized as follows: Section 2 formulates the coprime linear array data model. In Section 3, the proposed method based on MI-ESPRIT and LUT is outlined in detail. Section 4 provides the numerical simulations and the proposed algorithm is discussed referring to the simulation results. Section 5 concludes the paper.

Notations: Throughout this paper, we use lower-case letters and capital letters to represent the vector and matrix, respectively. Superscript ${\left(\cdot \right)}^{-1}$, ${\left(\cdot \right)}^{H}$, and ${\left(\cdot \right)}^{+}$ denote the inverse, conjugate transpose, and pseudo-inverse operator, respectively. The notation [*A*]*_ij_* represents the (*i*,*j*)th element of the matrix *A*. The symbol *E* denotes the statistical expectation operator and ‖·‖ is the Euclidean norm operator. arcsin(•) denotes the anti-sinusoidal operator and *I_M_* stands for the *M* × *M* identity matrix. Moreover, we use *R*(•) to represent the rounding operator.

## 2. CLA Data Model

**Figure 1:**
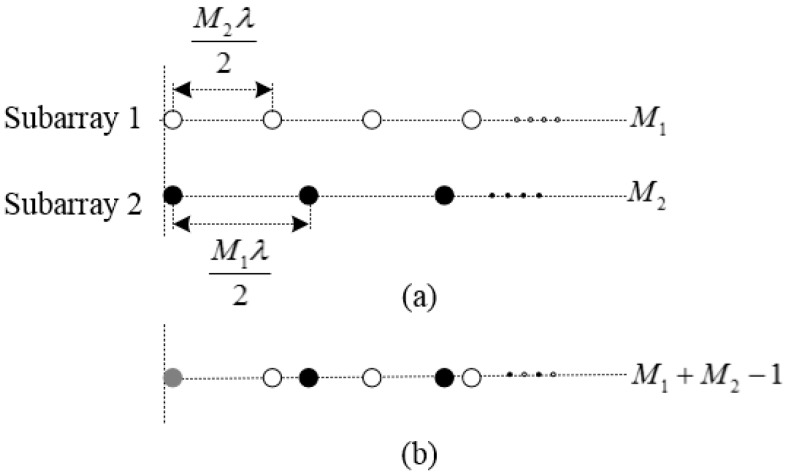
Topological structure of a coprime linear array (CLA): (**a**) Two uniform linear subarrays; (**b**) Coprime array formed by aligning the above two linear subarrays.

We considered a CLA consisting of two uniform linear subarrays. The number of array elements are $M_{1}$ and $M_{2}$, where $M_{1}$ and $M_{2}$ are the coprime integers. The inter-element spacings are $d_{1}=M_{2}\lambda /2$ and $d_{2}=M_{1}\lambda /2$, where λ is the signal wavelength, as shown in Figure 1a. By aligning the first elements of the two subarrays, we obtain the CLA, which includes $M_{1}+M_{2}-1$ elements, as shown in Figure 1b. Assuming that K far-field signals imping on the array from angles $\theta_{1},\theta_{2},\cdots ,\theta_{K}$.

Since the distance between adjacent elements is unequal, there is no general steering vector expression. So, we constructed the output vector model from the perspective of two subarrays. For the ith subarray with $M_{i}$ elements, the output vector can be modeled as:

(1) $$X_{i}(t)=A_{i}S(t)+N(t),$$

where S(t) is the signal vector and N(t) is the additive white Gaussian noise vector. The steering vector $A_{i}$ can be expressed as:

(2) $$A_{i}=[a_{i}(\theta_{1}),a_{i}(\theta_{2}),\cdots ,a_{i}(\theta_{K})].$$

The array manifold of the kth signal is:

(3) $$a_{i}(\theta_{k})=\left[1,e^{-j\frac{2\pi }{\lambda }d_{i}\sin(\theta_{k})},\cdots ,e^{-j\frac{2\pi }{\lambda }(M_{i}-1)d_{i}\sin(\theta_{k})}\right]{}^{T},$$

where $d_{i}$ is the inter-element spacing and i=1,2.

Then, we calculated the covariance matrix $R_{X_{i}X_{i}}$ of the output vector $X_{i}(t)$:

(4) $$R_{X_{i}X_{i}}=E\{X_{i}X_{i}{}^{H}\}=A_{i}R_{S}A_{i}{}^{H}+\sigma_{n}^{2}I_{i}.$$

The eigen-decomposition of $R_{X_{i}X_{i}}$ can be expressed as:

(5) $$R_{X_{i}X_{i}}=U_{S_{i}}\Sigma_{S_{i}}U_{S_{i}}{}^{H}+U_{N_{i}}\Sigma_{N_{i}}U_{N_{i}}{}^{H},$$

where $U_{S_{i}}$ is the signal subspace of the ith subarray.

## 3. Proposed Algorithm

### 3.1. DOA Estimation Based on the MI-ESPRIT Algorithm

**Figure 2:**
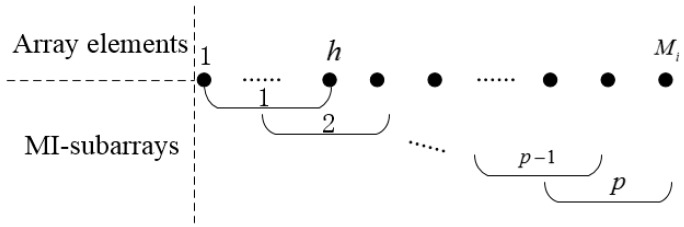
Array structure of the multiple invariance estimation of signal parameters via rotational invariance technique (MI-ESPRIT).

The ESPRIT algorithm achieves DOA estimation by taking advantage of only a single displacement invariance in the sensor array. However, there are many situations where the subarray possesses multiple invariance structures. In order to make full use of the displacement invariance of the subarray and improve angle measurement precision, we used the MI-ESPRIT algorithm to estimate DOAs for the CLA. According to the reference [29], in order to obtain the multiple invariance structures, the ith subarray is divided into p arrays, and each array owns h elements. There are h−1 overlapping elements between adjacent arrays. Therefore, the number of arrays and the number of array elements satisfy the following relationship:

(6) $$p+h-1=M_{i},$$

where $M_{i}$ denotes the number of the ith subarray’s elements, as shown in Figure 2.

The signal subspace $U_{S_{i}}$ and the steering vector of the ith subarray span the same space, i.e.,

(7) $$span\{U_{S_{i}}\}=span\{A_{i}(\theta )\}.$$

For the CLA, a unique non-singular matrix T exists:

(8) $$U_{S}=\left[\begin{array}{c} U_{S_{1}}\\ U_{S_{2}} \end{array}\right]=\left[\begin{array}{c} A_{1}(\theta )\\ A_{2}(\theta ) \end{array}\right]T.$$

According to the MI structure of the subarray, we constructed a singular value decomposition (SVD)-like matrix $E_{i}$

(9) $$E_{i}={\left[\begin{array}{cccc} U_{S_{i1}} & U_{S_{i2}} & \cdots & U_{S_{ip}} \end{array}\right]}^{T},$$

where $U_{S_{ij}}=U_{S_{i}}(j:j+h-1,:),(j=1,2,\cdots ,p)$ means extracting the jth row to the (j+h−1)th row of $U_{S_{i}}$ as a new matrix.

Define matrix $E_{i_{1}}$ and $E_{i_{2}}$ from $E_{i}$:

(10) $$E_{i_{1}}=\left[\begin{array}{c} U_{S_{i1}}\\ U_{S_{i2}}\\ \vdots \\ U_{Si(p-1)} \end{array}\right]=Q_{i_{1}}T,\;E_{i_{2}}=\left[\begin{array}{c} U_{Si2}\\ U_{Si3}\\ \vdots \\ U_{Sip} \end{array}\right]=Q_{i_{2}}T.$$

From Equation (10), we see that $Q_{i_{1}}$ and $Q_{i_{1}}$ exist in the following relationship:

(11) $$Q_{i_{2}}=Q_{i_{1}}\psi_{i},$$

where $\psi_{i}=diag(e^{-j\frac{2\pi }{\lambda }d_{i}\sin(\theta_{1})},e^{-j\frac{2\pi }{\lambda }d_{i}\sin(\theta_{2})},\cdots ,e^{-j\frac{2\pi }{\lambda }d_{i}\sin(\theta_{K})})$ is a diagonal matrix and $\psi_{i}$ contains the direction information of incoming signals. Thus, we obtain:

(12) $$E_{i_{2}}=Q_{i_{2}}T=Q_{i_{1}}\psi_{i}T=E_{i_{1}}T^{-1}\psi_{i}T.$$

Define $\Omega_{i}=T^{-1}\psi_{i}T$. Equation (12) can be modified as:

(13) $$\Omega_{i}=E_{i_{1}}{}^{+}E_{i_{2}},$$

where $E_{i_{1}}{}^{+}$ denotes the Moore-Penrose pseudo-inverse of $E_{i_{1}}$.

Since T is non-singular matrix, $\Omega_{i}$ and $\psi_{i}$ have the same eigenvalues. After completing eigen-decomposition on matrix $\Omega_{i}$, we obtain K eigenvalues $\lambda_{i_{1}},\lambda_{i_{2}},\cdots ,\lambda_{i_{K}}$. According to $\lambda_{i_{k}}=e^{-j2\pi d_{i}\sin(\theta_{k})/\lambda }$, the DOAs of K signals can be estimated:

(14) $${\hat{\theta }}_{i_{k}}=arcisn(angle(\lambda_{k})c/2\pi d_{i}f_{k}).$$

### 3.2. Solving Ambiguity Based on the LUT

Since the inter-element spacing of each subarray was larger than a half-wavelength, the DOA estimation results were ambiguous. In Section 3.1, for every incoming signal, only an estimated value was obtained based on the MI-ESPRIT algorithm. First, we needed to calculate all estimated values according to the coprime property, which includes real DOA and ambiguous DOA. Then, by using the solving ambiguity method, real DOA can be obtained. The earliest solving ambiguity method obtains the real DOA by searching the nearest value from all DOA estimated values of two subarrays, which is computationally complex. Additionally, matching errors may occur when the incoming wave contains multiple signals. The reference [27] proposed a search-free solving ambiguity method, but solving the modular equations requires an iterative process, which still requires considerable calculation. Therefore, a solving ambiguity method with ultra-high speed is urgently required. In engineering applications, we usually take advantage of the LUT method to reduce the computational complexity.

#### 3.2.1. Construct the LUT

For any signal, real DOA ${\hat{\theta }}_{r}$ and ambiguous DOA ${\hat{\theta }}_{a}$ satisfy:

(15) $$\sin({\hat{\theta }}_{r})-\sin({\hat{\theta }}_{a})=\frac{2l_{m}}{M},$$

where M is the number of subarray elements and $l_{m}$ is a non-zero integer number between −(M−1) and (M−1). For subarray 1, $M=M_{2}$. For subarray 2, $M=M_{1}$.

In order to simplify the procedure of constructing the LUT, we performed the transformation as $u=\sin(\hat{\theta }),-1\leq u\leq 1$. Thus, Equation (15) can be written as:

(16) $$u_{r}-u_{a}=\frac{2l_{m}}{M}.$$

In the transformation domain, it can be seen from Equation (16) that the estimated values are uniformly distributed.

(17) $$\{-1+(i-1)\frac{2}{M}\leq u\leq -1+i\frac{2}{M}\},(i=1,\cdots ,M).$$

By calculating Equations (14)–(17), we obtain all DOA estimation values of the two subarrays. According to the reference [23], real DOAs uniquely exist, which satisfies the estimated values of the two subarrays. We considered one signal impinging on the CLA. Denote $\left\{{\hat{\theta }}_{m_{1}}\right\}$ as the estimated values set of subarray 1 and denote $\left\{{\hat{\theta }}_{m_{2}}\right\}$ as the estimated values set of subarray 2, where $m_{1}=1,2,\cdots M_{2},\;m_{2}=1,2,\cdots ,M_{1}$. By solving the minimum of the following formula,

(18) $$\underset{m_{1},m_{2}}{\min}\left\|\left\{{\hat{\theta }}_{m_{1}}\right\}-\left\{{\hat{\theta }}_{m_{2}}\right\}\right\|,$$

we can obtain the real DOA:

(19) $$\hat{\theta }=\frac{{\hat{\theta }}_{m_{1}}+{\hat{\theta }}_{m_{2}}}{2}.$$

For the given CLA, the number of subarray elements is determined. In the transformation domain, the estimated values of subarray 1 are uniformly distributed in $\left\{-1+\frac{2}{M_{2}}(i-1)\leq u_{1}\leq -1+i\frac{2}{M_{2}}\right\}$, $i=1,\cdots ,M_{2}$ and those of subarray 2 are uniformly distributed in $\left\{-1+\frac{2}{M_{1}}(j-1)\leq u_{2}\leq -1+\frac{2}{M_{1}}j\right\}$, $j=1,\cdots ,M_{1}$.

**Figure 3:**
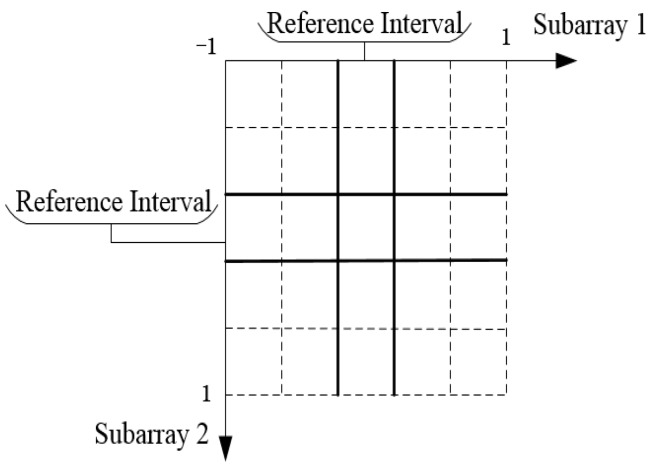
Constructing the lookup table (LUT).

For any incoming signal $\theta_{k}\in [-\pi /2,\pi /2]$, every subinterval always has an estimated value, and all DOA estimated values can be obtained according to any subinterval estimated value. Select any subinterval of two subarrays as the reference interval, as shown in the solid line of Figure 3. So, we chose the reference interval of the two subarrays in the transformation domain to construct the LUT. In the reference interval, we set $d_{s}$ as the step. The smaller the $d_{s}$, the higher the angle measurement precision. However, too small $d_{s}$ increases the size of the table, so we generally chose $d_{s}=0.01$ in practice. As the two subarrays traverse the entire reference interval at step $d_{s}$, we can obtain the corresponding incident angle:

(20) $$\begin{array}{l} \theta_{1}(I_{1})=\arcsin((I_{1}-1)d_{s})\\ \theta_{2}(I_{2})=\arcsin((I_{2}-1)d_{s}) \end{array},$$

where $I_{1}=1,\cdots ,(R(2/M_{2}d_{s})+1)$ and $I_{2}=1,\cdots ,(R(2/M_{1}d_{s})+1)$, R(·) represents the rounding symbol.

Firstly, the estimated values set to $\left\{\theta_{1}(I_{1})\right\}$ and $\left\{\theta_{2}(I_{2})\right\}$, which correspond to $\theta_{1}(I_{1})$ and $\theta_{2}(I_{2})$, are calculated based on Equations (16) and (17), respectively. Then, substituting $\left\{\theta_{1}(I_{1})\right\}$ and $\left\{\theta_{2}(I_{2})\right\}$ into Equations (18) and (19), respectively, we obtain the real DOAs corresponding to $\left\{\theta_{1}(I_{1})\right\}$ and $\left\{\theta_{2}(I_{2})\right\}$. Finally, the real DOA of each pair $I_{1}$ and $I_{2}$ is stored in the table. We then constructed the LUT by traversing $I_{1}$ and $I_{2}$. Since the LUT is only constructed in the reference interval, the table is smaller.

#### 3.2.2. Solving Real DOA Based on the LUT

The established LUT in the above section assumes there only one signal exists. When K(K≥2) signals impinge on the CLA, each subarray can have K estimated values based on MI-ESPRIT. However, there is no consistent one-to-one matching relationship between the estimated values of the two subarrays. In order to perform DOA estimation based on the above LUT, it was necessary to find the pairing relationship between the estimated values of the two subarrays.

This problem has been mentioned by the reference [25]. We defined $H_{1}$ and $H_{2}$ according to the signal subspace of the two subarrays.

(21) $$H_{1}=U_{S_{2}}U_{S_{1}}^{+}=A_{2}TT^{-1}A_{1}{}^{+},$$

(22) $$H_{2}=U_{S_{1}}U_{S_{2}}^{+}=A_{1}TT^{-1}A_{2}{}^{+},$$

which means

(23) $$A_{2}=H_{1}A_{1},$$

(24) $$A_{1}=H_{2}A_{2}.$$

Considering that K signals imping on the CLA from angles $\theta_{1},\theta_{2},\cdots ,\theta_{K}$. Based on the MI-Esprit algorithm, subarray 1 can obtain estimated values ${\hat{\theta }}_{1k}$ and subarray 2 can obtain estimated values ${\hat{\theta }}_{2k}$, where k=1,2,⋯,K. Taking the first estimated value ${\hat{\theta }}_{11}$ of ${\hat{\theta }}_{1k}$, we can obtain the corresponding array steering vector $a_{11}=\left[1,e^{-j\frac{2\pi }{\lambda }d_{1}\sin({\hat{\theta }}_{11})},\cdots ,e^{-j\frac{2\pi }{\lambda }(M_{1}-1)d_{1}\sin({\hat{\theta }}_{11})}\right]{}^{T}$. According to Equation (23), we know $a_{12}=H_{1}a_{11}$. Meanwhile, K estimated values of subarray 2 also correspond K array steering vectors $a_{21},a_{22},\cdots ,a_{2K}$. We can obtain matched ${\hat{\theta }}_{2k}$ by calculating the minimum

(25) $${\hat{\theta }}_{2k}=\underset{a_{12},a_{2k}}{\min}\left\|a_{12}-a_{2k}\right\|.$$

According to Equation (25), the remaining estimated values ${\hat{\theta }}_{12},\cdots ,{\hat{\theta }}_{1K}$ of subarray 1 correspond to the matched estimated values in subarray 2, which can also be solved. Finally, the pairwise combination of the two subarrays’ DOA estimated values can be achieved.

For any set of estimated values ${\hat{\theta }}_{1k}$ and ${\hat{\theta }}_{2k}$ of the two subarrays, we first calculated their corresponding $u_{1k}$ and $u_{2k}$ in the transformation domain, and then obtained their index values in the table.

(26) $$I_{1k}=\{\begin{array}{c} R(\frac{u_{1k}+1}{d_{s}})\;-1\leq u_{1k}\leq -1+\frac{2}{M_{2}}\\ \vdots \\ R(\frac{u_{1k}+\frac{2}{M_{2}}-1}{d_{s}})\;1-\frac{2}{M_{2}}\leq u_{1k}\leq 1 \end{array}\;I_{2k}=\{\begin{array}{c} R(\frac{u_{2k}+1}{d_{s}})\;-1\leq u_{2k}\leq -1+\frac{2}{M_{1}}\\ \vdots \\ R(\frac{u_{2k}+\frac{2}{M_{1}}-1}{d_{s}})\;1-\frac{2}{M_{1}}\leq u_{2k}\leq 1 \end{array}.$$

**Figure 4:**
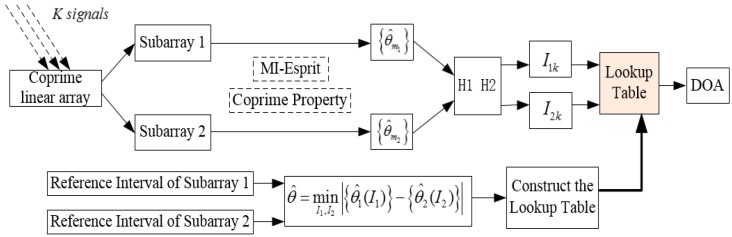
The calculation procedure of the proposed algorithm.

Substituting $I_{1k}$ and $I_{2k}$ into the LUT, the real DOA ${\hat{\theta }}_{k}$ can be solved. The complete calculation procedure of the algorithm is shown in Figure 4. The proposed method realizes DOA estimation of multiple signals, by only using one signal to construct the LUT, which is relatively simple and convenient for practical application. In practical engineering, the proposed algorithm in this paper could be implemented on the hardware platform composed of the digital signal processor (DSP) and field-programmable gate array (FPGA), where the LUT is built in the microwave darkroom in advance and stored in the RAM module of the FPGA chip.

## 4. Simulation Analysis

**Figure 5:**
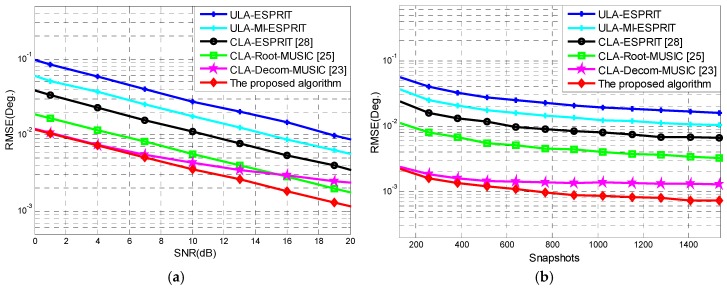
DOA estimation precision analysis: (**a**) Root mean square error (RMSE) of DOA estimation versus the signal-to-noise ratio (SNR), and (**b**) RMSE of DOA estimation versus snapshots.

Consider a CLA consisting of two uniform linear subarrays with $M_{1}=13$ and $M_{2}=11$ elements, and inter-element spacing of $d_{1}=M_{2}\lambda /2$ and $d_{2}=M_{1}\lambda /2$, respectively. In MI-ESPRIT, we take h=4, and then the subarray 1 can be divided into $p_{M_{1}}=10$ arrays and subarray 2 can be divided into $p_{M_{2}}=8$ arrays. Two signals imping on the CLA from −20°, 20°. In order to verify the performance of the proposed algorithm, we performed comparison simulations between the previously proposed methods [23,25,28] and our method. In the reference [23], the rough step was *d_S_*_1_ = 0.1° and the fine step was *d_S_*_2_ = 0.2°. Moreover, in order to illustrate the advantage of CLA, a comparison with the uniform linear array (ULA) was added to the simulation, in which the number of array elements was consistent with the CLA, i.e., $M_{1}+M_{2}-1$. We performed 1000 independent Monte Carlo simulations. We recorded 512 snapshots and Figure 5a shows that the DOA estimation root mean square error (RMSE) of the six algorithms varied with the signal-to-noise ratio (SNR). When the SNR=10 dB, the DOA estimation RMSE of the six algorithms versus snapshots is shown in Figure 5b.

With increasing SNR or snapshots, the DOA estimation RMSE of the algorithms decreased rapidly. Compared with the ULA, the DOA estimation precision of CLA was significantly better. Compared to both CLA and ULA, the DOA estimation precision of MI-ESPRIT was better than ESPRIT. This is because the MI-ESPRIT algorithm makes full use of the subarray elements, so the angle measurement precision of the proposed algorithm is better than that of the reference [28], which was based on the ESPRIT algorithm. The DOA estimation precision of the reference [25] was between that of the reference [28] and the proposed algorithm. It can be seen from Figure 5 that the DOA estimation precision of the algorithm introduced by the reference [23] is basically consistent with our proposed method when SNR is low. With increasing SNR, the DOA estimation precision of the algorithm of the reference [23] was poor compared to our proposed method. Because the angle measurement accuracy in the reference [23] is closely related to the precision of the fine spectrum search step *d_S_*_2_, if we continued to reduce *d_S_*_2_, the DOA estimation precision of the reference’s algorithm [23] may be better at high SNR, but the high computational complexity caused by the fine spectrum search would be unfeasible. In summary, the proposed algorithm possesses the best DOA estimation accuracy.

In order to verify the angular resolution of the proposed method, other simulation conditions remained unchanged and the DOAs of two signals were reduced to −10°, 10°; −5°, 5° and −0.5°, 0.5°. The SNR=5 dB, SNR=10 dB, and SNR=15 dB DOA estimation results are shown in Table 1. It can be seen from Table 1 that the proposed method could still distinguish two signals, although the DOA spacing was 1°. This is because the MI-ESPRIT algorithm has higher DOA estimation precision. Additionally, in the process of constructing the lookup table, we set the step $d_{s}=0.01$ in the reference interval, which guarantees high estimation accuracy. Higher DOA estimation precision means higher angular resolution. Therefore, the angular resolution of the proposed method can achieve 1°.

The computational complexity of the various algorithms are analyzed in Table 2. The CLA consists of two uniform linear subarrays with $M_{1}$ and $M_{2}$ sensors. Consider that K signals impinge on the CLA and T snapshots are used, and the number of searches in the reference [23] is set as S. It can be seen from Table 2 that the computational complexity of each algorithm is mainly created by two parts. The first part obtains the estimated values, which include covariance matrix estimation, eigenvalue decomposition, and solving estimated values, so the computational burden of the four algorithms is different. The second part is the solving ambiguity; the computational complexities in the previously formulated algorithms [23,25,28] are the same, i.e., $K^{2}M_{1}M_{2}$, which is larger than the complexity in our method. In the proposed algorithm, by simply indexing in the LUT, the real DOA can be obtained. In general, the computational burden of the first part is larger than the second part.

In the reference [23], the computational burden is caused by the spectrum peak search process and S is usually much larger than other variables, so CLA-Decom-MUSIC [23] has the highest computational complexity. CLA-Root-MUSIC [25] estimates the covariance matrix and performs the eigenvalue decomposition by combining the two subarrays, which increases the computational complexity, and the polynomial root finding is very time-consuming in practice. Therefore, CLA-Root-MUSIC [25] also has higher computational complexity, which second to CLA-Decom-MUSIC [23]. Both CLA-ESPRIT [28] and our proposed algorithm are essentially based on the ESPRIT algorithm, whose computational complexity is lower than CLA-Decom-MUSIC [23] and CLA-Root-MUSIC [25]. For the ESPRIT algorithm, because the computational burden of solving estimated values can be ignored, its computational burden of the first part mainly includes covariance matrix estimation and eigenvalue decomposition of both the covariance matrix $R_{X_{i}X_{i}}$ and matrix $\Omega_{i}$, and the corresponding complexities are $(M_{1}{}^{2}+M_{2}{}^{2})T$, $\left(M_{1}{}^{3}+M_{2}{}^{3}\right)$ and $(3(M_{1}+M_{2})+4K)K^{2}$, respectively. The MI-ESPRIT algorithm uses the multiple invariance structure of ESPRIT, which only adds the linear transformation of the matrix compared to ESPRIT. The added computational burden can also be neglected. Therefore, the proposed algorithm has approximately similar computational complexity in terms of the first part compared with CLA-ESPRIT [28]. However, we simplified the solving ambiguity by using a LUT, which substantially reduces the computational burden, i.e., $K\ll K^{2}M_{1}M_{2}$. Therefore, the proposed algorithm is more efficient.

**Figure 6:**
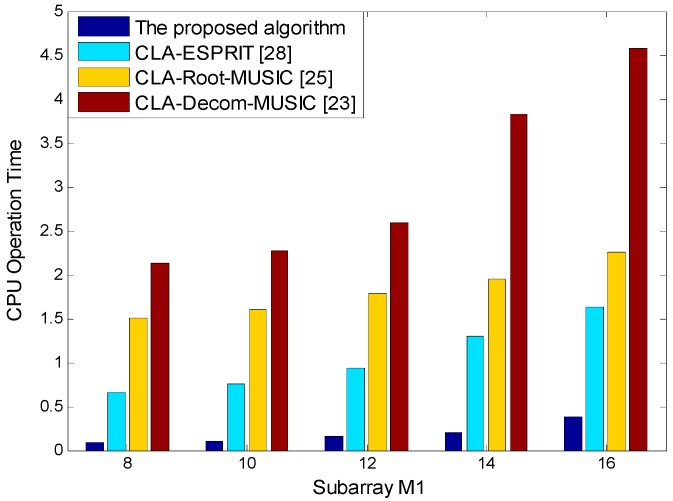
Computational complexity analysis.

According to the above simulation conditions, $M_{1}=13$ remains unchanged and $M_{2}$ is 8, 10, 12, 14, and 16. We compared the processing time of the four different algorithms in Figure 6. The processing time was determined by a PC (Lenovo manufactory, Beijing, China) with AMD Phenom™ IIX6 1055T Processor 2.8 GHz CPU and 8 GB RAM by running the MATLAB codes in the same environment. It can be seen from Figure 6 that the proposed algorithm has the highest computational efficiency.

## 5. Conclusions

CLA has been widely studied due to its superior DOA estimation performance. This paper proposed a novel DOA estimation algorithm for CLA based on MI-ESPRIT and LUT. MI-ESPRIT fully uses the subarray’s elements, which improves the angle measurement accuracy. Then, according to the property of the CLA, the phase ambiguity was solved using the LUT, which reduced the computational complexity. At the same time, using the relationship between the signal subspace of two subarrays, matching errors were avoided when in the presence of multiple signals. Compared with the existing algorithms, the proposed method not only has higher DOA estimation accuracy, but also has lower computational complexity. Additionally, our DOA estimation method, which is based on the LUT, has broad application prospects in practice. However, in our study, the coprime array achieved DOA estimation by decomposing CLA into two uniform linear subarrays, which sacrifices the degrees of freedom, i.e., a reduction in the number of sources that the CLA can resolve. By increasing the number of CLA sensors, we could obtain increased DOFs. In addition, the virtualization array sensor method, using the Khatri-Rao transformation, could also be applied to the CLA to yields the virtual array structure. Based on the extended virtual array aperture, the DOFs can also be increased. Therefore, future research efforts will aim to improve the DOF of CLA.

## Figures and Tables

**Table 1:** The angular resolution analysis of the proposed method.

	*θ*_1_ = −10°	*θ*_2_ = 10°	*θ*_1_ = −2°	*θ*_2_ = 2°	*θ*_1_ = −0.5°	*θ*_2_ = 0.5°
SNR = 5 dB	−9.9934°	10.0055°	−1.9952°	2.0019°	−0.4965°	0.4918°
SNR = 10 dB	−9.9991°	9.9992°	−1.9992°	1.9997°	−0.4996°	0.5011°
SNR = 15 dB	−10.0002°	10.0000°	−2.0000°	2.0004°	−0.5002°	0.4999°

**Table 2:** Comparison of computation complexity.

Algorithm	Computation Complexity
CLA-Decom-MUSIC [23]	$O((M_{1}{}^{2}+M_{2}{}^{2})T+(M_{1}{}^{3}+M_{2}{}^{3})+(M_{1}{}^{2}/M_{2}+M_{2}{}^{2}/M_{1})S+K^{2}M_{1}M_{2})$
CLA-Root-MUSIC [25]	$O({\left(M_{1}+M_{2}\right)}^{2}T+{\left(M_{1}+M_{2}\right)}^{3}+(M_{1}{}^{3}+M_{2}{}^{3})+K^{2}M_{1}M_{2})$
CLA-ESPRIT [28]	$O((M_{1}{}^{2}+M_{2}{}^{2})T+(M_{1}{}^{3}+M_{2}{}^{3})+(3(M_{1}+M_{2})+4K)K^{2}+K^{2}M_{1}M_{2})$
The proposed method	$O((M_{1}{}^{2}+M_{2}{}^{2})T+(M_{1}{}^{3}+M_{2}{}^{3})+(3(M_{1}+M_{2})+4K)K^{2}+K)$
